# Repetitive Transcranial Magnetic Stimulation of the Primary Motor Cortex beyond Motor Rehabilitation: A Review of the Current Evidence

**DOI:** 10.3390/brainsci12060761

**Published:** 2022-06-10

**Authors:** Abdulhameed Tomeh, Abdul Hanif Khan Yusof Khan, Liyana Najwa Inche Mat, Hamidon Basri, Wan Aliaa Wan Sulaiman

**Affiliations:** 1 Department of Neurology, Faculty of Medicine and Health Sciences, Universiti Putra Malaysia, Serdang 43400, Malaysia; abdulhameedtomeh5@gmail.com (A.T.); ahanifkhan@upm.edu.my (A.H.K.Y.K.); liyananajwa@upm.edu.my (L.N.I.M.); hamidon@upm.edu.my (H.B.); 2 Malaysian Research Institute on Ageing (MyAgeingTM), Universiti Putra Malaysia, Serdang 43400, Malaysia

**Keywords:** transcranial magnetic stimulation, therapeutic use, primary motor cortex, non-motor symptoms

## Abstract

Transcranial magnetic stimulation (TMS) has emerged as a novel technique to stimulate the human brain through the scalp. Over the years, identifying the optimal brain region and stimulation parameters has been a subject of debate in the literature on therapeutic uses of repetitive TMS (rTMS). Nevertheless, the primary motor cortex (M1) has been a conventional target for rTMS to treat motor symptoms, such as hemiplegia and spasticity, as it controls the voluntary movement of the body. However, with an expanding knowledge base of the M1 cortical and subcortical connections, M1-rTMS has shown a therapeutic efficacy that goes beyond the conventional motor rehabilitation to involve pain, headache, fatigue, dysphagia, speech and voice impairments, sleep disorders, cognitive dysfunction, disorders of consciousness, anxiety, depression, and bladder dysfunction. In this review, we summarize the latest evidence on using M1-rTMS to treat non-motor symptoms of diverse etiologies and discuss the potential mechanistic rationale behind the management of each of these symptoms.

## 1. Introduction

The primary motor cortex (M1) consists of a population of neurons that play a crucial role in the voluntary regulation of movement [1]. Transcranial magnetic stimulation (TMS) was introduced to study the human M1 in 1985 as a novel, painless technique that can be delivered non-invasively [2]. Application of TMS in a repetitive manner can induce neuroplastic effects in the targeted region and its functionally connected networks and thus alter neuronal excitability beyond the period of stimulation [3]. Conventional repetitive TMS (rTMS) protocols in research and clinical practice include high-frequency (HF)-rTMS (5–20 Hz) and low-frequency (LF)-rTMS (≤1 Hz), which can increase or decrease M1 excitability, respectively, for several minutes after stimulation [4,5]. Another rTMS protocol, known as theta-burst stimulation (TBS), was developed later with reduced administration duration. The TBS protocol consists of extremely high-frequency (50 Hz) stimulation in the pattern of three bursts at the theta range (5 Hz) [6]. This protocol can be applied as intermittent TBS (iTBS) or continuous TBS (cTBS), which have comparable efficacy to HF-rTMS and LF-rTMS, respectively [6]. The impact of HF-rTMS/iTBS on enhancing M1 excitability and LF-rTMS/cTBS on reducing M1 excitability is thought to rely on principles of long-term potentiation (LTP) and long-term depression (LTD) plasticity, respectively [7]. At the cellular level, LTP/LTD plasticity results from a prolonged strengthening/inhibition of synaptic transmission following synchronous/asynchronous presynaptic and postsynaptic activity [8].

To localize the primary motor cortex (M1), a single-pulse TMS is applied away from the vertex towards the right or left M1 to activate the motor neurons and induce a muscle twitch. This twitch can be measured by electromyography (EMG) to record the motor evoked potential (MEP). Originally, a “motor hotspot” was defined as the optimal TMS coil position over M1 that evokes MEPs of maximum amplitude and shortest latency in a target muscle [9]. However, due to practical issues, the motor hotspot is more commonly localized as the TMS coil position over M1 that evokes the largest and most consistent MEP amplitude from a target muscle, regardless of its latency [10,11]. In some cases, because of stroke or corticospinal tract injury, MEPs might be absent upon M1 stimulation. Still, the motor hotspot can be targeted using the mirror image of the unaffected hemisphere [12]. Another method to localize the M1 in TMS studies is by magnetic resonance imaging (MRI) based on specific anatomical landmarks of the M1, i.e., hand knob [13], or by functional MRI (fMRI) while performing a specific motor task [14]. However, the motor hotspot method is more commonly employed in the TMS literature [15].

Afterward, the motor threshold is measured to personalize the TMS intensity for each individual. Resting motor threshold (RMT) is defined as the lowest TMS intensity needed to evoke an MEP of ≥50 μV in 5 of 10 consecutive trials in the relaxed muscle. In comparison, active motor threshold (AMT) is the lowest TMS intensity required to elicit MEP ≥200 μV in 5 of 10 consecutive trials during an isometric contraction of the target muscle of 10–20% of its maximal strength [3]. In therapeutic applications, the TMS intensity is usually reported as a percentage of the RMT in conventional rTMS paradigms, and AMT in studies that employ TBS paradigms [15].

Following localization of the motor hotspot and determining the TMS intensity, the TMS coil is fixed over the M1 for the whole treatment session. Jung et al. found that marking the hotspot with a felt-tip pen yielded similar consistency of MEPs compared with using a neuronavigation system with MRI guidance [16]. Nonetheless, expert panels advise using a neuronavigation system to ensure higher accuracy while applying the TMS coil over the M1 [15]. Concerning the types of the TMS coils, a figure-of-eight coil is most commonly applied at the hand and face regions of the M1 as it produces focal and superficial stimulation. While non-focal coils, such as H-coils and double-cone coils, are used preferably to target the lower limb and pelvic representation of the M1 as it produces deeper stimulation [17].

As a therapeutic approach, recent meta-analyses have shown that M1-rTMS is a promising intervention for motor recovery in Parkinson’s disease [18], multiple sclerosis [19], and post-stroke rehabilitation [20]. However, apart from the motor responses, M1-rTMS has shown a potential to treat a variety of symptoms, including pain, headache, fatigue, dysphagia, speech and voice impairments, sleep disorders, cognitive dysfunction, disorders of consciousness, anxiety, depression, and bladder dysfunction. In this review, we aim to summarize the current evidence on using rTMS over M1 to treat non-motor symptoms in a “symptomatic” rather than “syndromic” approach.

## 2. Materials and Methods

The Medical Subject Heading (MeSH) database of PubMed was searched in March 2022 for the MeSH term “transcranial magnetic stimulation” and subheading term “therapeutic use”. Titles and abstracts of the search results were screened to identify the type of symptoms that have been treated with M1-rTMS across patient populations, and non-motor symptoms were extracted. These symptoms included pain, headache, fatigue, bladder dysfunction, dysphagia, speech and voice impairments, anxiety, depression, cognitive dysfunction, sleep disorders, and disorders of consciousness.

A subsequent search in the PubMed database was performed using the following search syntax: (“transcranial magnetic stimulation”[tiab] OR TMS[tiab] OR rTMS[tiab] OR “theta burst”[tiab] OR TBS[tiab]) AND (“motor cort*”[tiab] OR M1[tiab]) AND (pain[tiab] OR painful[tiab] OR headache[tiab] OR migraine*[tiab] OR fatigue*[tiab] OR bladder[tiab] OR urin*[tiab] OR dysphagia[tiab] OR swallow*[tiab] OR aphasia[tiab] OR dysarthria[tiab] OR speech[tiab] OR voice[tiab] OR anxiety[tiab] OR mood[tiab] OR depressi*[tiab] OR cogniti*[tiab] OR dementia[tiab] OR sleep*[tiab] OR conscious*[tiab]).

Articles published in English and related to therapeutic M1-rTMS were extracted, and the references were further checked for additional relevant studies. Case reports and studies that included M1 stimulation as part of a multisite stimulation protocol were excluded from this synthesis.

The hierarchy of the current evidence is presented with a particular focus on the latest expert guidelines and systematic reviews and meta-analyses that have conducted subgroup analysis on the M1-rTMS therapeutic efficacy. When none of these sources were identified, individual studies were succinctly described.

Notably, the vast majority of M1-rTMS studies in the reviewed literature were sham-controlled studies and applied a therapeutic protocol of 5 daily sessions/week of high-frequency (≥5 Hz), subthreshold intensity (<100% RMT), using a figure-of-eight coil. Therefore, these will be the conventional stimulation parameters in this synthesis unless stated otherwise.

## 3. Pain

Over the years, various brain stimulation techniques at M1 have been trialed with a promising analgesic efficacy, including invasive epidural motor cortex stimulation [21] and non-invasive techniques such as transcranial direct current stimulation (tDCS) [22] and rTMS [23].

The rationale behind the analgesic efficacy of M1 stimulation relies mainly, but not exclusively, on its interconnections with the endogenous opioid system. Positron emission tomography (PET) scans demonstrated that M1 stimulation directly potentiated the top-down opioid-mediated inhibition system [24,25]. In addition, blocking μ-opioid receptors with the drug naloxone significantly reduced the analgesic efficacy of M1 stimulation [26], further supporting the relation between M1-rTMS stimulation and the release of endogenous opioids. Therefore, recent evidence highlights the potential role of blood β-endorphin measurement as an objective response biomarker in treating chronic pain with rTMS [27,28].

Another putative mechanism pertains to the glutamate receptor, N-methyl-D-aspartate (NMDA). The drug ketamine, an NMDA receptor antagonist, significantly reduced the analgesic efficacy of high-frequency rTMS over M1, suggesting a shared pathway with the LTP-like plasticity mechanisms [29]. In addition, a disruption in the γ-aminobutyric acid (GABA)-mediated intracortical inhibition was noticeable in both acute [30] and chronic pain conditions [31]. In turn, high-frequency M1-rTMS was shown to restore the defective intracortical inhibition with a direct correlation between the analgesic effect and cortical excitability [32,33,34]. This notion highlights the principle of state dependency of TMS, where the facilitatory effect of high-frequency rTMS is reversed and the cortical excitability decreases if the high-frequency rTMS is applied during a state of enhanced cortical excitability [35,36].

On the neural network level, neuroimaging studies have shown that M1 stimulation modulated the excitability of other cortical and subcortical areas related to sensory, cognitive, and emotional components of pain, such as the thalamus, insular cortex, and anterior cingulate gyrus [37,38].

### 3.1. Chronic Pain

The International Association for the Study of Pain (IASP) defines chronic pain as pain that persists or recurs for more than three months [39]. The IASP task force has recently developed a pragmatic classification comprising seven groups to unify the reporting of chronic pain conditions [39]. This classification will be followed in this review except for the headache conditions, which will be discussed separately.

#### 3.1.1. Chronic Neuropathic Pain

Neuropathic pain (NP) is pain caused by a lesion or disease of the somatosensory system, leading to pain hypersensitivity and spontaneous pain [40]. NP can be classified as central or peripheral based on the site of etiology [40]. 

Recent systematic reviews and meta-analyses have converged on the analgesic efficacy of M1-rTMS in various NP conditions. These conditions included both central NP, such as central post-stroke pain, and peripheral NP, such as trigeminal neuralgia, post-herpetic neuralgia, painful radiculopathy, and diabetic polyneuropathy [41,42,43,44,45]. The most common TMS parameters that yielded analgesic effects were: a stimulation frequency of 10–20 Hz, stimulation intensity of 80–120% RMT, number of pulses of 1000–2000, and for 5–10 sessions [44], with 5 sessions producing analgesia that can sustain for at least one month [45].

In early meta-analyses, the differential analgesic effect of rTMS was thought to depend on the neuroanatomical origin of NP, in that patients with central NP would respond better than peripheral NP conditions [45,46]. However, a recent meta-analysis has challenged this assumption showing no significant difference in the analgesic efficacy of rTMS between central and peripheral NP [43].

Another critical factor to consider is whether the M1 stimulation site must correspond to the somatotopy of the painful body region [47,48]. Nonetheless, accumulating evidence supports the notion that M1-rTMS alleviates pain in a non-somatotopic manner. For instance, rTMS stimulation of the M1 hand representation caused pain reduction in different body areas in NP [49] and non-NP conditions such as endometriosis [50] and low-back pain [51].

A recent Cochrane meta-analysis demonstrated that low-frequency rTMS and rTMS applied over the dorsolateral prefrontal cortex (DLPFC) were ineffective for pain reduction. On the other hand, high-frequency M1-rTMS exerted minor, short-term analgesic effects, yet negligible to be clinically significant [52]. However, Cochrane reviews attribute a low risk of bias only for studies with a follow-up duration of more than eight weeks and recruiting more than 200 participants per arm [52], a condition rarely fulfilled in the TMS literature. Still, two separate groups of expert panels in Europe and North America have recently granted a level A evidence (definite efficacy) for the high-frequency M1-rTMS contralateral to the painful side in NP management [15,53]. The recommended treatment protocol for NP consisted of 5–10 induction sessions (at >24 and <72 intervals), stimulation frequency at 10–20 Hz with an intensity of 80–90% RMT, and a total of 2000–3000 pulses per session. In the absence of comorbid severe depression, the contralateral M1 is the target for unilateral NP and the left DLPFC is the target for diffuse NP. While in the presence of comorbid severe depression along with NP, the left DLPFC is targeted with the induction sessions increased to more than 10 sessions. The maintenance sessions consist of biweekly to monthly sessions based on the treatment benefits [53].

#### 3.1.2. Chronic Primary Pain

Chronic primary pain (CPP) is defined as pain in one or more anatomical regions associated with emotional distress and significant functional disability (i.e., interfering with daily activities) that last longer than three months and cannot be better accounted for by another diagnosis [54]. CPP comprises chronic widespread pain, complex regional pain syndrome, chronic primary visceral pain, chronic primary musculoskeletal pain, and chronic primary headache or orofacial pain [54].

##### Chronic Widespread Pain

Chronic widespread pain is defined as diffuse pain in more than four body regions in more than three body quadrants, along with the core features of CPP [55]. Fibromyalgia and myofascial pain syndromes were the main conditions studied in the M1-rTMS literature under this category.

Fibromyalgia syndrome (FMS) is a severe widespread pain associated with sleep disorders and cognitive dysfunction [54]. Two recent meta-analyses concluded that high-frequency rTMS over the left M1 effectively relieved pain and improved the quality of life in patients with FMS [56,57]. The improvement in FMS symptomology was associated with changes in resting-state connectivity and metabolism in sensory, affective, and cognitive brain processing areas, as shown in fMRI [58] and PET scans [59]. Furthermore, delivering maintenance sessions for >13 weeks is thought to sustain the analgesic effects of rTMS among these patients [57]. Nonetheless, expert panels have recently granted a level B evidence (probable efficacy) for the high-frequency rTMS over the left M1 for improving quality of life and over the left DLPFC for analgesia in FMS patients [15].

On the other hand, myofascial pain syndrome (MPS) is defined as pain in the muscles and surrounding fascia with the presence of hypersensitive sites known as “trigger points” [60]. As addressed in the recent systematic review of Yang et al., 10 sessions of high-frequency rTMS over the left M1 was considered a beneficial therapeutic option for patients with chronic MPS [61]. However, no expert recommendations exist for M1-rTMS in this category due to the paucity of data.

##### Complex Regional Pain Syndrome

Complex regional pain syndrome (CRPS) is a posttraumatic pain, mostly in distal extremities, that is disproportionate in duration and magnitude to any inciting event [62]. Applying 10 sessions of high-frequency rTMS at M1 contralateral to the painful limb significantly alleviated pain in patients with CRPS [61] and was granted a level C (possible efficacy) based on the latest experts’ consensus [15].

##### Chronic Primary Visceral Pain

Chronic primary visceral pain comprises CPP syndromes that affect the digestive system’s viscera in the head/neck and thoracic region and the digestive, urinary, and genital systems’ viscera in the abdomen [54].

In patients with irritable bowel syndrome (IBS), applying 5 sessions of rTMS over the left M1 hand region did not significantly reduce abdominal pain intensity but improved the maximum rectal tolerated volume in a subgroup of IBS patients with marked hypersensitivity [63]. While applying a single session of rTMS over the M1 anorectal region increased the rectal sensory threshold in IBS patients but not healthy volunteers [64]. On the other hand, patients with bladder pain syndrome/interstitial cystitis experienced a significant improvement in pain intensity for 6 weeks following 10 sessions of rTMS at high-frequency, suprathreshold intensity (110% RMT), using an H-10 coil over the M1 pelvic region [65].

##### Chronic Primary Musculoskeletal Pain

Chronic primary musculoskeletal pain is a CPP that involves muscles, bones, joints, or tendons [54]. Of this category, 5 sessions of rTMS over the right M1 hand region significantly alleviated the pain levels in patients with non-specific low back pain [51]. Moreover, applying 8 maintenance sessions on weeks 3, 4, 6, 8, 12, 20, 28, and 36 sustained the analgesic effects during this period [51]. On the other hand, an ongoing study is investigating whether delivering 3 daily sessions followed by 7 weekly maintenance sessions of rTMS at the M1 hand region combined with motor control exercise can yield an additive analgesic efficacy in patients with chronic low back pain in comparison to either rTMS or exercise alone [66].

#### 3.1.3. Chronic Secondary Musculoskeletal Pain

Secondary musculoskeletal pain is defined as musculoskeletal pain resulting from a disease classified elsewhere [67]. In a study of musculoskeletal pain related to Parkinson’s disease, 5 sessions of rTMS at the M1 hand region, contralateral to the painful side or over the left M1 in diffuse pain, significantly alleviated the motor symptoms and musculoskeletal pain for up to 1 month [68]. While in patients with secondary progressive multiple sclerosis and spasticity-associated pain in the lower limbs, 10 sessions of bilateral M1-rTMS, but not iTBS, significantly decreased the pain levels for at least 2 weeks after the stimulation course [69]. Another study from this category was conducted in patients with post-stroke hemiplegic shoulder pain associated with adhesive capsulitis [70]. In this study, 10 sessions of rTMS at the M1 hand region of the affected hemisphere reduced shoulder pain significantly for 1 month but did not improve the joint range of motion during this period. Finally, a single non-shamcontrolled study explored the analgesic efficacy of M1-rTMS in patients with human T-lymphotropic virus type 1 (HTLV-1)—associated myelopathy with painful spasticity [71]. In their study, 5 sessions of rTMS at the right M1 leg region significantly decreased the pain levels for 1 week and spasticity for 1 month after treatment.

#### 3.1.4. Chronic Secondary Visceral Pain

The IASP defines chronic secondary visceral pain as a chronic pain secondary to an underlying condition originating from internal organs of the head/neck, thoracic, abdominal, and pelvic regions [72]. Of this category, two non-shamcontrolled studies were reported on two distinct gender-related morbidities. In patients with endometriosis, 5 sessions of rTMS at the left M1 hand region improved the pain intensity, pain interference, and gastrointestinal quality of life for 1 month [50]. While in patients with chronic prostatitis/chronic pelvic pain syndrome, delivering 5 sessions of suprathreshold rTMS (110% RMT) at the bilateral M1 pelvic regions was well-tolerated and reduced the pain intensity for 2 months after treatment [73].

#### 3.1.5. Chronic Postsurgical or Posttraumatic Pain

According to the IASP classification, chronic pain after surgery or trauma should be separately categorized as postsurgical or posttraumatic pain, even if neuropathic mechanisms are crucial in their etiology [74].

Of this category, phantom limb pain (PLP) is defined as the perception of pain in a missing body part, mostly occurring in the distal portion of a missing limb [74]. Based on the results of two recent meta-analyses involving three M1-rTMS studies, the analgesic efficacy of 5 sessions of both high-frequency or low-frequency rTMS over M1 contralateral to the amputated limb was non-significant in patients with PLP [43,75]. Notably, previous TMS studies in PLP were mostly motor mapping studies to elaborate on the pathophysiology of this condition. These studies have shown that PLP was associated with M1 reorganization in the affected hemisphere. However, this reorganization did not correlate with the level of PLP perception [76].

Whereas in chronic pain after spinal cord injury, two recent meta-analyses involving six studies on high-frequency M1-rTMS reported a significant analgesic effect of this technique [43,77], mainly in the middle term (2–6 weeks post-treatment) rather than the early term (about one-week post-treatment) [77]. However, the number of sessions in their analysis was heterogeneous, ranging from 1 to 10 treatment sessions [43,77].

On the other hand, three individual studies reported a significant analgesic efficacy for at least one month after 10 sessions of M1-rTMS in patients with failed back surgery syndrome [78] and chronic central pain after traumatic brain injury [79], and after 5 sessions in patients with traumatic brachial plexus injury [80].

Noteworthy, some authors have recently argued about the potential of M1-rTMS to reduce the transition from acute to chronic pain by breaking down the chain reactions that lead to pain chronification if applied during the acute stages in traumatically injured patients [81]. This theoretical framework is indeed worth investigating in future clinical trials.

#### 3.1.6. Chronic Cancer-Related Pain

Even though chronic cancer-related pain was considered earlier as nociceptive pain, due to tumor compression, or neuropathic pain, as a result of cancer treatment, the IASP indicates that both should be regarded as one category [82]. In patients with various types of malignancies and pain locations, 10 sessions of rTMS at the hand region corresponding to the painful side significantly reduced the pain for 2 weeks and then returned to baseline levels at 1-month post-treatment [83]. Additionally, a recent pilot study investigated the analgesic efficacy of different coil directions over M1 in patients with chemotherapy-induced peripheral neuropathy [84]. A single session of lateral-medial (LM) and the conventional posterior-anterior (PA) coil directions over the M1 target extremity had a significant analgesic effect and reduced dysesthesia for one hour after stimulation.

### 3.2. Acute Pain

The analgesic efficacy of M1-rTMS has been less investigated in acute pain conditions compared to the aforementioned intractable chronic pain syndromes. Furthermore, most rTMS studies on acute pain were in healthy participants following experimental induction of pain perception. However, the experimental pain is beyond the scope of this article, which only focuses on the clinical use of M1-rTMS among patient populations for the purpose of treatment.

Acute neuropathic pain has been the primary investigated condition of this category. Zhao et al. showed that 6 sessions/week for 3 weeks of M1-rTMS significantly reduced the pain levels within the first week only in acute neuropathic pain after spinal cord injury [85], and until the end of the third week of treatment in acute central poststroke pain [86]. In both studies, serum levels of brain-derived neurotrophic factor (BDNF) remained significantly elevated until the end of the third week of treatment [85,86]. BDNF is one of the most studied neurotrophins, a family of polypeptides that promote neuronal survival and synaptic transmission and plasticity [87]. Some authors proposed using BDNF serum levels as a biological marker for the therapeutic use of rTMS in depression and post-stroke rehabilitation [28]. However, the evidence that BDNF might play a role in the analgesic response to rTMS is scarce.

### 3.3. Headache

Even though headache and scalp discomfort are among the adverse effects of TMS stimulation [17], this technique is still grabbing attention in headache management as treatment protocols are being further optimized. The International Headache Society (IHS) classifies headache disorders into three categories comprising primary headaches, secondary headaches, and painful cranial neuropathies and facial pain [88].

#### 3.3.1. Primary Headaches

Recent systematic reviews and meta-analyses have converged on the promising contribution of M1-rTMS in reducing headache frequency, intensity, duration, abortive medication use, and functional impairment in both episodic and chronic migraine [89,90,91,92,93]. The preferred TMS parameters in the meta-analysis of Moisset et al. were high-frequency (10 Hz) rTMS applied over the left M1, with 600–3000 pulses per session, stimulation intensity at 70–80% RMT, and for 1–10 sessions [91]. In addition, some studies demonstrated that the clinical improvement in headache frequency correlated with the plasma β-endorphin levels after rTMS [94,95]. Of note, the food and drug administration (FDA) has recently approved the application of single-pulse TMS over the occipital cortex for the acute and preventive treatment of migraine [96]. However, rTMS application over M1 is still not approved for either of these indications. On the other hand, expert panels have recently granted the same moderate level of evidence for both high-frequency M1-rTMS and occipital single-pulse TMS for migraine prevention [53]. Nonetheless, due to the heterogeneity between previous studies, the IHS has recently issued guidelines to optimize the design and conduct of controlled trials on neuromodulation devices, including TMS, for the treatment of migraine [97].

On the other hand, while tension-type headache (TTH) is the most prevalent neurological disorder worldwide [98], the TMS literature related to this condition is scarce. In a single study by Kalita and colleagues, both single and three sessions of rTMS applied at the left M1 hand region were equally effective in reducing headache frequency, intensity, and abortive medication use for three months in patients with chronic TTH [99].

Similar to TTH, cluster headache was rarely investigated in the TMS literature, presumably due to the lesser disabling impact of these two conditions compared to migraine. In a single non-sham controlled study, Hodaj et al. reported a cumulative analgesic effect of rTMS at the M1 face region, contralateral to the painful side when it was applied as 10 sessions, followed by biweekly, then 5 monthly maintenance sessions in patients with cluster headache [100].

#### 3.3.2. Secondary Headaches

The literature on M1-rTMS highlights posttraumatic brain injury-related headache (PTBI-HA) as the main investigated condition among secondary headaches, and the therapeutic indication of M1-rTMS in this condition was granted a high recommendability by expert panels [53]. The recommended treatment protocol for PTBI-HA consisted of 5 induction sessions (at >24 and <72 intervals), stimulation frequency at 10–20 Hz with an intensity of 80–90% RMT, and a total of 2000–3000 pulses per session. In the absence of comorbid severe depression, either left M1 or left DLPFC are chosen as cortical targets. In the presence of comorbid severe depression, the left DLPFC is targeted with the induction sessions increased to more than 10 sessions. The maintenance sessions consist of biweekly to monthly sessions based on the treatment benefits [53].

#### 3.3.3. Painful Cranial Neuropathies and Other Facial Pain Conditions

Of note, many conditions of this category can be classified with double parenting in chronic neuropathic pain based on the IASP classification, such as trigeminal neuralgia (TGN) and facial post-herpetic neuralgia (PHN) [40]. Nonetheless, two recent systematic reviews have shown a promising analgesic potential of M1-rTMS in chronic orofacial pain conditions, including TGN, PHN, and persistent idiopathic facial pain (formerly known as atypical facial pain) [101,102]. Moreover, despite the facial localization of pain, rTMS at the M1 hand region was more favored than the M1 facial region for pain reduction in these conditions [101]. In addition, more sessions at the high-frequency stimulation and more pulses per session appeared to exert longer analgesic effects [101]. Expert panels have strongly recommended using high-frequency M1-rTMS to treat neuropathic pain, including TGN and PHN [53], with the same stimulation parameters as discussed earlier.

## 4. Fatigue

Fatigue is a frequent and disabling symptom experienced in various diseases and cannot be completely explained by conventional structural damage [103]. With the lack of effective treatments, M1-rTMS has been applied to relieve fatigue in patients with fibromyalgia syndrome, multiple sclerosis, amyotrophic lateral sclerosis, and chronic neuropathic pain. The mechanism of action of rTMS in fatigue management remains unknown. However, applying rTMS at M1 might modulate the functional connectivity between the impaired neural networks in these patients, resulting in reduced fatigue perception [104].

In two recent meta-analyses, fatigue severity scales (FSS) did not show significant improvement following rTMS in fibromyalgia syndrome (FMS) patients [56,57]. However, neither of these studies conducted a subgroup analysis on the effect of targeting M1 and only included FSS as an outcome measure for fatigue, which was used in a single M1-rTMS study. On the contrary, an earlier meta-analysis performed a subgroup analysis on the M1-rTMS effect on fatigue and analyzed different fatigue scales, including a total of eight M1-rTMS studies [105]. In conclusion, they found that M1-rTMS significantly reduced the fatigue levels in FMS patients in comparison to sham stimulation [105]. While stimulation parameters were heterogeneous, most studies in their analysis applied the conventional parameters, i.e., high-frequency, subthreshold intensity, using a figure-of-eight coil over the left M1.

On the other hand, in patients with relapsing-remitting multiple sclerosis (RR-MS)-related fatigue, Gaede et al. reported safety and a significant reduction in fatigue levels following 3 sessions/week over 6 weeks of bilateral M1 simulation using the deep H10 coil [104]. This improvement in fatigue remained significant for another 6 weeks after treatment. In another RR-MS study, 10 sessions of M1-iTBS contralateral to the affected leg and combined with exercise therapy significantly reduced the spasticity and fatigue by the end of the treatment course in comparison to either iTBS or exercise alone [106]. While in patients with secondary-progressive multiple sclerosis (SP-MS), 10 sessions of bilateral M1-rTMS, but not iTBS, significantly decreased the fatigue levels for at least 2 weeks after the stimulation course [69]. Meanwhile, both rTMS and iTBS improved spasticity levels for the 2-week duration following treatment.

While in a single study of amyotrophic lateral sclerosis, 10 sessions of high-frequency, suprathreshold (110% RMT), bilateral M1-rTMS were applied using a figure-of-eight coil over the upper limbs’ and circular coil over the lower limbs’ M1 representation sequentially in patients with ALS [107]. A significant improvement in fatigue and quality of life was observed by the end of treatment, but these effects were no longer evident 2 weeks later.

Finally, scores of chronic fatigue significantly improved following the application of 5 or 15 sessions of rTMS, and to a lesser extent iTBS, at M1 contralateral to the painful side in patients with chronic NP-associated fatigue [49,108].

## 5. Dysphagia

The rationale behind employing M1-rTMS in dysphagia management relies mainly on its effect on the corticobulbar projections to swallowing muscles [109]. Applying rTMS over the swallowing musculature hotspot at M1 has been trialed with promising results in dysphagia after stroke, Parkinson’s disease, and in the context of aging, aka presbydysphagia.

Recent systematic reviews and meta-analyses have converged on the efficacy of M1-rTMS for improving swallowing function in post-stroke dysphagia [110,111,112,113]. This therapeutic efficacy was better achieved with M1-rTMS than with other non-invasive neurostimulation techniques, namely transcranial direct current stimulation, surface neuromuscular electrical stimulation, and pharyngeal electrical stimulation [110,112]. Stimulation targets of the rTMS varied between pharyngeal, esophageal, mylohyoid, and tongue cortical areas of the M1, and the number of sessions ranged from 1–10 sessions [111]. The effects were most substantial when rTMS was applied during the first two weeks after stroke and within the first two months following its application [111]. In addition, bihemispheric M1-rTMS showed better effects on swallowing recovery than ipsilesional and contralesional M1-rTMS [111]. However, there is an ongoing debate about the superiority of high-frequency vs. low-frequency stimulation and targeting ipsilesional vs. contralesional M1 in unilateral stimulation [112]. Therapeutic guidelines by expert panels recommend applying M1-rTMS in post-stroke dysphagia as an adjunct to swallowing training with a moderate level of evidence [114].

On the other hand, two individual studies found that 10 sessions of bilateral M1-rTMS significantly improved the swallowing function in patients with Parkinson’s disease (PD)-associated dysphagia [115,116], and enhanced activation of the caudate nucleus and parahippocampal gyrus in fMRI imaging [116]. Moreover, adding 5 maintenance sessions per month over 3 months sustained the swallowing improvement during this period [115].

Finally, two M1-rTMS studies were conducted in patients with presbydysphagia, a common swallowing difficulty in the geriatric population. In these studies, 10 sessions of high-frequency rTMS and 5 sessions of iTBS, both applied at the right M1, improved the swallowing function in elderly patients with dysphagia [117,118]. The improvement in swallowing after iTBS remained significant for at least 3 months post-treatment, regardless of the presence or absence of concomitant neurological diseases [118].

## 6. Speech and Voice Impairments

As M1 receives input from Broca’s area and projects through corticobulbar tracts to the muscles responsible for speech production [119], several clinical studies have investigated the potential to increase or decrease excitability in these tracts through rTMS protocols.

In patients with post-stroke non-fluent aphasia, a single session of low-frequency rTMS over the right M1 mouth region did not increase the pictures named [120,121,122] but led to a significant decrease in the response time, defined as the time between picture presentation and beginning the production of the correct name [121]. However, apart from the right M1 mouth region, a recent iTBS-fMRI study proposed targeting the affected M1 at the hand representation in patients with post-stroke aphasia [123]. These findings were consistent with previous reports showing functional connectivity between the M1 hand region and the cortical language network [124,125].

Whereas in patients with post-stroke dysarthria, 10 sessions of low-frequency rTMS over the contralesional M1 mouth region combined with speech therapy improved the articulation performance significantly in comparison to speech therapy alone [126].

In addition, M1-rTMS has been investigated in Parkinson’s disease (PD)—associated speech disorders. In hypokinetic dysarthria, for instance, a single session of high-frequency rTMS over the left M1 mouth region significantly improved the voice intensity, speech rate, and fundamental frequency [127,128] but did not affect the speech fluency and articulation [129]. Whereas targeting the M1 hand area, contralateral to the patients’ most affected side, did not improve the speech and voice in PD patients with no or minimal dysarthria [130]. On the other hand, 4 sessions/week for 4 weeks of high-frequency rTMS at the right or left M1 laryngeal region combined with intensive voice treatment did not affect the intonation significantly compared to intensive voice treatment alone in PD-associated hypophonia [131].

Lastly, apart from stroke and PD, M1-rTMS has been investigated in speech disorders associated with Tourette syndrome and adductor laryngeal dystonia. In Tourette syndrome, due to the involvement of M1 in the pathophysiology of this condition [132], some studies have applied a variety of rTMS parameters over the left M1, i.e., high-frequency vs. low-frequency, at subthreshold vs. suprathreshold intensity, and for a single session vs. 5 daily sessions [133]. However, none of these protocols significantly ameliorated the vocal tics associated with this syndrome, and the supplementary motor area is still considered the preferred target in these patients [133]. While in a different vocal pathology, a recent pilot study demonstrated that a single session of low-frequency rTMS applied over the left M1 laryngeal region was safe and feasible and improved the voice quality and perturbation in patients with adductor laryngeal dystonia [134]. A unique methodology to the latter study was the use of 90% of the cortical silent period, a measure of intracortical inhibition, as rTMS intensity instead of the conventional percentage of motor threshold [134].

## 7. Sleep Disorders

The therapeutic application of M1-rTMS in sleep disorders has been investigated in restless legs syndrome, sleep bruxism, obstructive sleep apnea, and sleep disturbances associated with neurological conditions, in particular Parkinson’s disease and chronic pain.

Restless legs syndrome (RLS) is characterized by M1 disinhibition and CNS dopaminergic dysfunction [135]. In turn, high-frequency rTMS at M1 was shown to restore the intracortical inhibition since its mechanism is state-dependent on the cortical excitability prior to TMS stimulation [35,36]. In addition, M1-rTMS activates the corticostriatal projections leading to the endogenous release of dopamine [136]. Applying 14 sessions of high-frequency rTMS to the left or right M1 leg region improved the motor features, anxiety, and subjective sleep quality in RLS patients [135] and was granted a level C evidence (weak evidence of effectiveness) based on expert guidelines [137].

In obstructive sleep apnea (OSA), while consecutive single-pulse TMS during sleep at the M1 submental region improved airflow dynamics, high-frequency rTMS protocols during either sleep or wakefulness caused detrimental upper airway response with a transient decrease in respiratory flow [138]. A presumable mechanism for this response was the disruption in the sequential physiological activation of upper airway dilators induced by rTMS, along with a concurrent stimulation of constrictor muscles [139,140].

While in sleep bruxism, a single pilot study reported that 5 sessions of low-frequency, bilateral rTMS at the M1 masseter muscle representation improved the muscle soreness and inhibited the jaw-closing muscle activity during sleep for 5 days post-treatment [141].

On the other hand, the effects of rTMS on sleep disturbances in neurological and psychiatric conditions have been addressed in the recent systematic review of Babiloni and colleagues [142]. In their review, non-M1 regions (i.e., DLPFC and parietal cortex) were the most frequent regions targeted in sleep disturbances, including primary insomnia. However, M1-rTMS for sleep disturbances has been mainly employed in Parkinson’s disease (PD) and chronic pain conditions where sleep quality is frequently deteriorated [142]. The mechanistic rationale was predicated on reducing the nocturnal recurrence of motor symptoms and pain in PD and chronic pain conditions, respectively, resulting in indirect improvement in sleep quality. On the other hand, inducing LTP-like plasticity in the M1 during wakefulness was found to modulate the slow-wave activity during sleep and could thereby regulate the sleep need [143,144]. In chronic pain conditions, 10 sessions of high-frequency unilateral M1-rTMS yielded analgesic efficacy and improved subjective measures of sleep quality [142]. Whereas in PD patients, 10 sessions of high-frequency, bilateral M1-rTMS improved subjective and objective measures of sleep quality in some [145,146], but not all studies [147,148]. These heterogeneous results might be explained by the difference in rTMS parameters (e.g., suprathreshold >100% RMT [145,146] vs. subthreshold <100% RMT intensities [147,148]) and the different outcome measures (e.g., polysomnography [146] vs. actigraphy [147]).

## 8. Cognitive Dysfunction

To date, the vast majority of non-invasive brain stimulation studies have targeted the DLPFC as a therapeutic approach in patients with cognitive dysfunction [149,150]. Besides, as M1-rTMS studies have mainly investigated its therapeutic efficacy on non-cognitive symptoms, patients with cognitive dysfunction were usually excluded from these studies as they might affect the subjective outcome measures and confound the interpretation of the results. Nonetheless, M1-rTMS has been investigated in a few studies of affected cognition in Parkinson’s disease (PD), stroke, and fibromyalgia syndrome. The procognitive effect of M1 stimulation in these conditions can be rationalized by the increasingly identified roles of M1 in higher cognitive processes, such as attention, memory, motor imagery, and language comprehension, and the functional connectivity between M1 and parietal cortex that supports the planning and execution of goal-oriented movements [151,152,153,154,155].

In patients with PD-associated dementia, applying 10 sessions of bilateral M1-rTMS at the hand region significantly improved the measures of global cognition and reduced the latency of P300, an event-related potential (ERP) marker of cognitive processing speed by the end of the treatment course. However, adding 5 maintenance sessions per month for 3 months did not preserve the initial cognitive improvement, which returned to the pre-treatment values by the end of the study [156]. Whereas in PD patients with freezing of gait, applying 5 sessions of unilateral rTMS over the M1 leg region did not yield procognitive effects unless it was combined with tDCS stimulation over the DLPFC in which the domain of executive function significantly improved [157].

On the other hand, in patients with chronic stroke, applying 10 sessions of low-frequency rTMS over the contralesional M1 hand region significantly improved global cognition [158] and visuospatial recall memory [159] and reduced the latency of N200 and P300 markers of ERP, reflecting an improvement in perceptual and cognitive processing [159]. While Xu et al. observed that immediately after delivering iTBS at the ipsilesional M1 hand region, fMRI metrics demonstrated consistent effects around language and cognition-related regions, i.e., central opercular cortex, paracingulate gyrus, and superior parietal lobule in patients with post-stroke aphasia [123]. In view of this, we argue against using M1-rTMS as a control stimulation condition in cognitive research but rather as an active comparator.

Finally, even though cognitive dysfunction is a frequent complaint in patients with fibromyalgia syndrome (aka, fibrofog) [160], it was rarely addressed in previous M1-rTMS studies, which have mainly focused on the pain and mood symptoms of this syndrome. Nonetheless, Baudic et al. reported a significant improvement in attention and executive functions following 14 sessions of rTMS over the left M1 hand region (5 daily induction sessions, and 3 weekly then 3 fortnightly followed by 3 monthly maintenance sessions) [161].

## 9. Disorders of Consciousness

The mechanistic rationale for the therapeutic use of M1-rTMS in disorders of consciousness is related in part to EEG findings showing that high-frequency rTMS at M1 transiently increased neuronal oscillations in the α and β frequency [162]. In addition, fMRI studies demonstrated that high-frequency rTMS at M1 induced blood-oxygenation-level-dependent (BOLD) changes both locally and in remote brain regions, including the supplementary motor area, dorsal premotor area, putamen, cingulate motor area, and crucially, the thalamus [163].

In a recent meta-analysis, O’Neal and colleagues reported that rTMS was utilized without any adverse effects in patients with disorders of consciousness, including minimally conscious state (MCS) and vegetative state/unresponsive wakefulness syndrome [164]. Based on the individual patient data involved in their meta-analysis, O’Neal et al. reported a 30.4% response rate following high-frequency rTMS at M1, with the response being defined as any change in the Coma Recovery Scale-Revised (CRS-R) score above a patient’s baseline. The more remarkable improvement in CRS-R scores was associated with MCS diagnosis, an etiology of stroke or intracranial hemorrhage, receiving 10 or more sessions of rTMS, and if the rTMS treatment was initiated within the first three months from inciting injury [164].

## 10. Anxiety and Depression

In the M1-rTMS literature, anxiety and depression were mainly investigated as secondary outcomes to account for their potential confounding effect on the study results. In addition, cases of severe anxiety or depression were conventionally excluded from these studies as they might affect the interpretation of the results. This might explain, in part, the absence of clear efficacy of M1-rTMS as most patients had mild or no comorbid anxiety and depression, making it less likely to show a significant therapeutic effect. Intriguingly, however, two recent studies have investigated the effect of M1-rTMS in patients with stroke- and Parkinson’s disease-related depression and revealed antidepressant efficacy in these patients [148,165]. On the one hand, the mechanistic rationale behind the improvement in anxiety and depression following M1-rTMS might stem from its effect on the concomitant symptoms and consequently emotional improvement in mood and general behavior. On the other hand, a recent meta-analysis combined with resting-state fMRI reported a positive correlation between M1 and depressive disorders-regions of interest (ROI), hence proposing M1 as a potential therapeutic target in depressive disorders [166]. In addition, M1-rTMS stimulation was found to alter the serum levels of kynurenine [165,167], a tryptophan metabolite and one culprit in the pathophysiology of depression [168]. Indeed, there have been preliminary reports of antidepressant efficacy of M1-rTMS in patients with Parkinson’s disease, stroke, and chronic pain. However, these findings need to be replicated in larger randomized controlled studies.

In the study of Makkos et al. [148], the antidepressant efficacy of M1-rTMS was explored as a primary objective in PD patients with mild to moderate depression. As a result, 10 consecutive sessions (including weekend days) of bilateral M1-rTMS at the hand representation significantly improved the depressive symptoms for at least one month after treatment. While in a study of PD-related musculoskeletal pain and anxiety and depression, 5 sessions of unilateral M1-rTMS at the hand region significantly improved the scores of each of these symptoms for at least one month of follow-up [68]. In other studies, however, a protocol of a single session [169], 3 sessions [170], or 5 sessions/week for 2 weeks [146,147,171] of bilateral M1-rTMS did not improve the mood symptoms in PD patients.

On the other hand, in two non-shamcontrolled studies in stroke patients, a single session of 6 Hz primed- low frequency (1 Hz) rTMS at the contralesional M1 hand region significantly improved the depression scores for 5 days after stimulation [172]. While delivering 2 sessions/day for 22 total sessions of low-frequency rTMS at the contralesional M1 hand region and combined with motor rehabilitation showed a significant antidepressant efficacy and increased the serum kynurenine/tryptophan ratio by the end of the study [165]. Interestingly, these results were only noticeable when the low-frequency rTMS was applied to the right, but not the left, M1 cortex [165]. Noteworthy, the laterality of the antidepressant efficacy of rTMS has already been established based on the frequency of stimulation over right vs. left DLPFC [173]. However, the influence of rTMS frequency and laterality of stimulation on depression severity needs to be further investigated with the other depression-related ROIs [166], including M1.

Apart from stroke and PD, the antidepressant efficacy of M1-rTMS was also explored in chronic pain conditions. In the recent systematic review of Jiang et al., 11 studies investigated the effect of M1-rTMS on neuropathic pain-comorbid depression. Among them, only two studies reported a significant antidepressant efficacy following M1-rTMS in patients with chronic spinal cord injury and failed back surgery syndrome [43]. While in patients with fibromyalgia syndrome (FMS), two recent meta-analyses showed conflicting results regarding the effect of rTMS on anxiety and depression [56,57]. However, neither of these studies conducted a subgroup analysis for the effect of targeting M1 on anxiety and depression. Whereas, an earlier meta-analysis revealed that M1-rTMS exerted no significant effect on anxiety and depression compared to sham stimulation [105]. Nonetheless, the latest expert guidelines recommend delivering rTMS over DLPFC instead of M1 in cases of pain and headache with comorbid severe depression [53].

Finally, as anxiety represents a core feature of restless legs syndrome, M1-rTMS was effective in decreasing anxiety levels in these patients (discussed under sleep disorders).

## 11. Bladder Dysfunction

The mechanistic rationale for the use of M1-rTMS in this condition relies on its influence on the corticospinal tract excitability and consequently detrusor/urethral sphincter functionality [174]. In addition, recent evidence has revealed an essential role of certain M1 neuronal subpopulations in issuing the “order” to initiate voiding via their projections to the pontine micturition center [175,176]. To our knowledge, there have been three studies thus far that investigated the therapeutic efficacy of M1-rTMS on bladder dysfunction, of which 1 was sham-controlled [65] and 2 were non-sham controlled studies [174,177].

In patients with MS-associated bladder dysfunction, 10 sessions of high-frequency, threshold intensity (100% RMT) of rTMS at the M1 leg region significantly improved the obstructive symptoms of the voiding phase without affecting the irritative symptoms of the filling phase [174]. These effects were noticeable during the clinical and urodynamic evaluation 3 days after treatment.

While in patients with PD-associated bladder dysfunction, 10 sessions of low-frequency, subthreshold intensity of rTMS at the M1 pelvic floor region significantly improved the bladder capacity and irritative symptoms without affecting the obstructive symptoms of the voiding phase [177]. These effects remained significant during the follow-up period for 2 weeks post-treatment.

**Figure 1:**
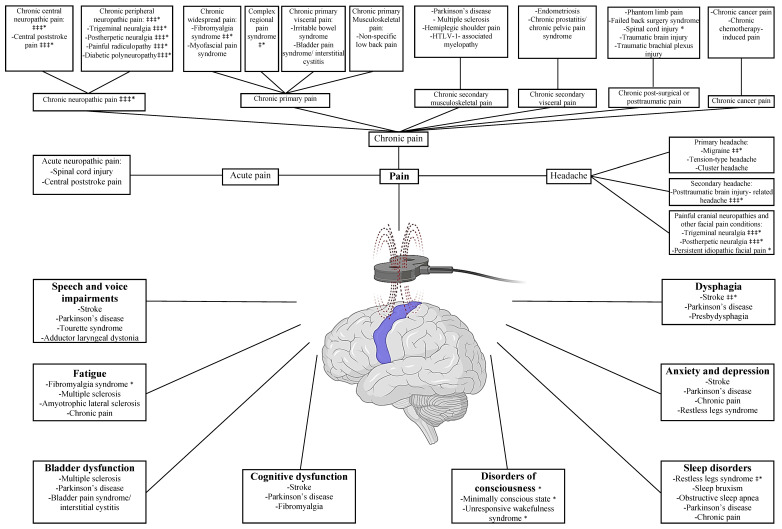
Graphical abstract summarizing the non-motor symptoms treated with M1-rTMS. ‡‡‡ denotes strong recommendation by current therapeutic guidelines, ‡‡ denotes moderate recommendation by current therapeutic guidelines, ‡ denotes weak recommendation by current therapeutic guidelines, * denotes supported by systematic reviews/meta-analyses. Figure created with BioRender.com (accessed on 19 November 2021).

Finally, in a sham-controlled study, a protocol of 10 sessions of high-frequency, suprathreshold intensity (110% RMT) using an H-10 coil over the M1 pelvic region was investigated in patients with bladder pain syndrome/interstitial cystitis [65]. A significant improvement occurred with respect to pain, urinary frequency, bladder emptying, nocturia, and urge incontinence that lasted for 6 weeks after treatment. A graphical abstract summarizing the pain and headache classification can be found in Figure 1.

## 12. Conclusions

The primary motor cortex (M1) represents the first and most studied brain region using TMS. This stems from the clear behavioral response following its stimulation, represented by muscle twitching that can be recorded via electromyography, and anatomical landmark on neuroimaging, represented by the hand knob. As anticipated, targeting M1 to treat motor symptoms (e.g., rigidity in Parkinson’s disease, spasticity in multiple sclerosis, post-stroke hemiplegia) has shown promising results in the TMS literature, which is reasonable due to the central role of M1 in the voluntary regulation of movement. However, with the advent of structural, functional, and molecular neuroimaging techniques, the cortical regions are being re-evaluated as hubs in a complex neurocircuitry inside the brain and thus an entry door for TMS into larger neural networks. Targeting M1 to treat non-motor symptoms has been increasingly investigated in the past few years, including pain (of neuropathic and non-neuropathic origin such as headache), fatigue, dysphagia, speech and voice impairments, sleep disorders, cognitive dysfunction, disorders of consciousness, anxiety, depression, and bladder dysfunction. 

Expert panels have recently granted a level A/strong evidence for applying high-frequency M1-rTMS in the treatment of chronic neuropathic pain and posttraumatic brain injury-related headache, a level B/moderate evidence in the migraine prevention and improving the quality of life in fibromyalgia, and a level C/weak evidence in the treatment of complex regional pain syndrome and restless legs syndrome [15,53,137]. In addition, M1-rTMS as an adjunct to conventional treatments was granted a moderate level of evidence for post-stroke dysphagia rehabilitation [114]. However, the experts’ consensus on the applicability of M1-rTMS in other non-motor symptoms is still not reached to date due to the scarcity of data. In addition, despite the clear benefit of M1-rTMS on certain chronic pain conditions, it is recommended to change the cortical target from M1 to DLPFC in the presence of comorbid severe depression [53].

Nonetheless, well-conducted randomized controlled trials, in particular studies combining TMS with EEG and neuroimaging techniques, will help expand our knowledge of the cortical and subcortical connections of M1 in health and disease and ultimately tailor the therapeutic use of rTMS based on the constellation of symptoms in each patient.

Moreover, while applying weekly to monthly maintenance sessions has shown promising preliminary results in the chronic pain literature, we believe that the influence of these sessions on other health conditions is worth investigating in future clinical studies. By doing so, these studies must first ensure the safety of delivering such extended sessions and then explore their efficacy in sustaining the rTMS effects among patients who respond to the initial induction sessions. 

Meanwhile, as standard pairwise meta-analyses provide high-quality evidence in clinical practice, an emerging method for comparative effectiveness research, known as network meta-analysis, has been increasingly used in the past decade to estimate the hierarchy and ranking in the effectiveness of more than one treatment protocol [178,179]. Such network meta-analyses are much needed in the brain stimulation field to compare the various TMS frequencies, intensities, and paradigms, the TMS and other brain stimulation techniques, such as tDCS, and the stimulation of M1 and other cortical regions, such as DLPFC, based on a shared clinical outcome.

In the end, we hope the synthesis of this review will aid investigators who are planning future trials on TMS and shed light on the clusters of symptoms that can be affected by M1-rTMS, in addition to the conventional motor symptoms, with different mechanistic rationales involving multiple neural networks.

## Data Availability

Not applicable.
